# Lemierre's syndrome resulting from streptococcal induced otitis media and mastoiditis: a case report

**DOI:** 10.1186/1752-1947-3-6658

**Published:** 2009-04-03

**Authors:** Che M Harris, Micean Johnikin, Hope Rhodes, Theresa Fuller, Sohail R Rana, Hasan Nabhani, Dulara Hussain, Mohan Kurukumbi, Annapurni Jayam-Trouth, Jhansi Gajjala, Faria Farhat, Vinod Mody

**Affiliations:** 1 Howard University Hospital, 2041 Georgia Avenue, NW, Washington, DC 20060, USA

## Abstract

**Introduction:**

Lemierre's syndrome is an extremely rare and almost universally fatal disease characterized as thrombophlebitis of the internal jugular venous system with subsequent metastatic infection. *Fusobacterium necrophorum* is the most common organism implicated in causation of Lemierre's syndrome. Group A *Streptococcus* has mainly been observed as a polymicrobial organism in the syndrome. We report a rare finding of a rare disease where *Group A Streptococcus* was the sole organism triggering Lemierre's syndrome. To our knowledge, this is only the third recorded patient with such an occurrence.

**Case presentation:**

We describe a 9-year-old African American boy, who presented with otitis media and mastoiditis that culminated in Lemierre's syndrome. Isolates bore only Group A *Streptococcus*. The patient was appropriately treated and responded with full recovery from the syndrome.

**Conclusion:**

Since Lemierre's syndrome is classically detected by clinical diagnosis, these findings should prompt clinicians to consider Group A *Streptococcus* as an alternative catalyst. It should be pondered that patients who present with typical Group A streptococcal infections have the possibility for developing Lemierre's syndrome. Though this complication appears to be rare, early diagnosis and prompt intervention have proven critical in survival outcome. Indeed, what would seem to be a routine case of strep throat or otitis media easily treated with antibiotics could end up being an unalterable progression to death unless Lemierre's syndrome is immediately diagnosed and treated.

## Introduction

Lemierre's syndrome, also known as necrobacillosis, and postanginal sepsis, was first described by Courmont, Cade, Goodman, Mosher and Scottmuller in the early 1900s [1]. In 1936, however, Andre Lemierre characterized 20 cases of oropharyngeal infections that were followed by anaerobic sepsis [2]. Hence, the syndrome, Lemierre's was born. During the pre-antibiotic era, the syndrome almost always resulted in death, with mortality as high as 90% [3]. It is an extremely rare condition with the number of reported cases noted to be about one per million per year [4]. Patients with Lemierre's syndrome usually present with systemic findings within 1 week after the onset of the inciting oropharyngeal infection. This is typically followed by ipsilateral neck pain and swelling [5]. A minority will have trismus or evidence of a thrombosed jugular vein on examination [5]. Baig *et al*. noted that Lemierre's syndrome typically follows a stepwise pattern in presentation [6]: Step I involves a precipitant primary infection, which may include fever, pharyngitis, otitis media, mastoiditis and parotitis. Step II is described as local microbial invasion to the lateral pharyngeal space and internal jugular vein from infected peritonsillar tissue via lymphatics; and Step III is characterized as septic metastatic spread.

## Case presentation

A 9-year-old mildly obese boy with a medical history significant for recurrent otitis media presented with sudden, one day, onset of sharp right-sided neck pain and neck stiffness that awakened him from sleep. He rated the pain 10 out of 10 in intensity, and noted it to be non-radiating, without alleviating or aggravating factors.

He admitted to subjective fever 1 week before presentation, associated neck stiffness, a frontal headache, right otalgia, pharyngitis and dysphagia. He also admitted to a single episode of vomiting clear fluid 1 day before experiencing the neck pain. He denied rash, tinnitus, odynophagia, cough, dyspnea, recent travel or exposure to sick contacts. However, 2 weeks before this presentation, he was treated with antibiotic eardrops for an episode of otitis media. The patient had no known drug allergies. His immunizations were up to date, and he was not taking any medications at the time of presentation.

In the emergency room, his temperature was 100.8°F, blood pressure 103/66, heart rate was 103, and respiratory rate at 22. On examination, he was a young, mildly obese boy in moderate distress, with no rash or skin lesions. His face was plethoric with tenderness to palpation appreciated along the right side of the sternocleidomastoid. Cervical lymphadenopathy was noted on palpation in this region of the neck. There was diminished range of motion on turning his neck to the right, secondary to pain. Kernig's and Brudzinski's signs were negative. No oropharyngeal erythema or oral lesions were noted. The right ear and mastoid were tender to palpation, and bilateral middle ear effusions were appreciated. He was tachycardic with regular rhythm, clear breath sounds, and normal abdominal exam. He was alert and oriented to person, place, and time, and cranial nerves II-XII were grossly intact. He had no focal neurologic deficits.

White blood cell count was 12,000/mm^3^, neutrophils 81.6%, lymphocytes 10%, monocytes 7.6%, eosinophils 0.3%, platelets 236 TH/CM, hemoglobin 11.3gm/dL, hematocrit 34.1% and erythrocyte sedimentation rate was 58mm/hour.

The patient was initially started on a combination regimen of clindamycin and ceftriaxone.

**Figure 1:**
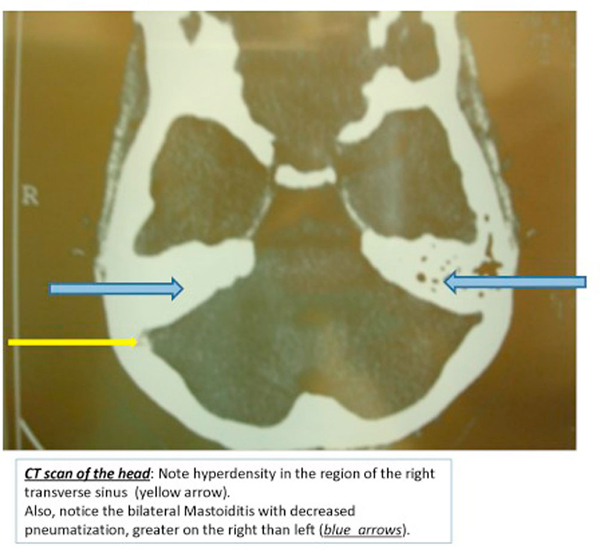
**Computed tomography scan of the head: Note hyperdensity in the region of the right transverse sinus (black arrow)**. Also, notice the bilateral mastoiditis with decreased pneumatization, greater on the right than left (white arrows).

**Figure 2:**
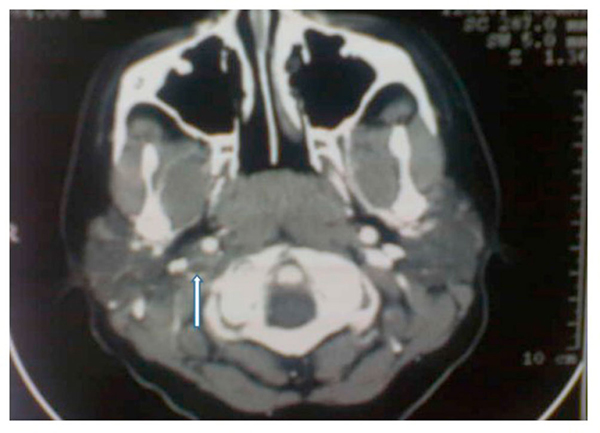
**Computed tomography scan of the head: Right internal jugular venous thrombosis (white arrow)**.

**Figure 3:**
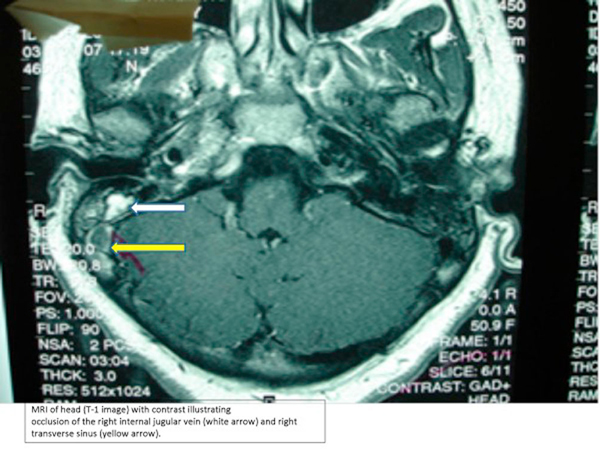
**Magnetic resonance imaging of the head: Right jugular venous thrombosis (white arrow) and right transverse sinus thrombosis (yellow arrow)**.

**Figure 4:**
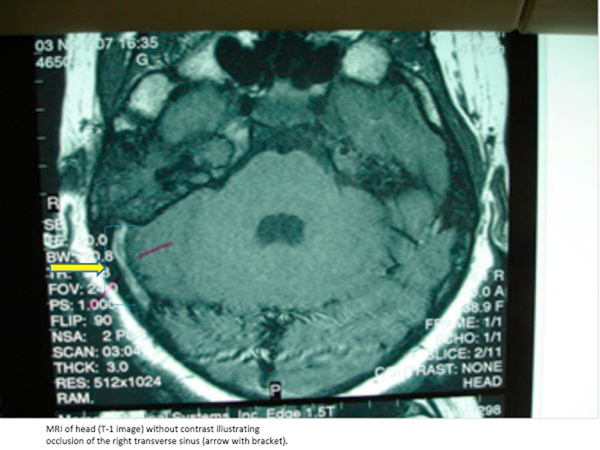
**Magnetic resonance imaging of the head: Cord sign of the right transverse sinus (yellow arrow with bracket)**.

**Figure 5:**
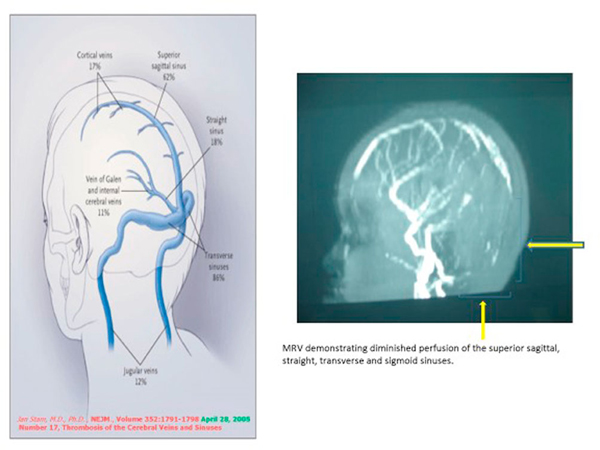
**Magnetic resonance venography of the head: Diminished perfusion of the superior sagittal, straight, transverse and sigmoid sinuses (brackets)**.

Computed tomography (CT) scan of the head demonstrated hyperdensity in the region of the right transverse sinus, and bilateral mastoiditis with decreased pneumatization, greater on the right than left (Figure 1). Further sections of head CT scan and magnetic resonance imaging (MRI) showed thrombosis of the right internal jugular vein (Figures 2 and 3). The classic cord sign, illustrative of transverse venous thrombosis, was also demonstrated via MRI (Figure 4). Magnetic resonance venography (MRV) demonstrated diminished perfusion of the superior sagittal, straight, transverse and sigmoid sinuses (Figure 5). The patient was subsequently started on therapeutic lovenox. Ceftriaxone was discontinued secondary to an allergic reaction.

Given the patient's predisposition for recurrent otitis media, prophylactic bilateral myringotomy and tympanostomy tubes were placed.

The patient improved significantly, with complete resolution of symptoms, and he was treated as an outpatient on therapeutic enoxaparin at 65mg subcutaneously every 12 hours for a duration of 4-6 weeks and oral clindamycin 300mg three times a day for a duration of 4 weeks. At follow-up, the patient was completely asymptomatic, and appeared to have recovered without any residual effects.

## Discussion

The most commonly implicated organism of Lemierre's syndrome based on culture results, mainly from blood has been the anaerobic Gram-negative rod, *Fusobacterium necrophorum* at 71.2% [6]. Up to 10.1% of reported cases consisted of *F. necrophorum* in combination with another organism [6]. About 5.5% of cases have involved an organism other than *F. necrophorum*[6]. Most notably, however, 12.8% of cases grew negative cultures [6]. In our patient, blood cultures were negative. However, throat culture revealed facultative anaerobic *Streptococcus pyogenes*, a rare cause of Lemierre's syndrome [6]. To our knowledge, only two other cases of Lemierre's syndrome caused solely by Group A *Streptococcus* have been recorded [7,8]. More importantly, before these findings, *Streptococcus pyogenes* had only been implicated twice as a polymicrobial organism in cases of Lemierre's syndrome, which strongly suggests its rarity as a cause of this disease [6,7].

This paper denotes a critical finding, and may suggest a new trend of rare catalysts of Lemierre's syndrome. Cultures negative for fusobacterium do not always rule out Lemierre's, and cultures positive for *Streptococcus pyogenes* do not rule out the possibility for developing the syndrome. Moreover, infections with *Streptococcus pyogenes* are typically viewed as easily treatable with appropriate antibiotics. However, we suggest that Lemierre's syndrome should be entertained in patients with suggestive symptoms, because unrecognized, it is almost always fatal. As in this patient, an early clinical diagnosis of Lemierre's syndrome was made, and confirmed by imaging studies. Our patient presented and was treated at Step II of the pathogenesis of Lemierre's syndrome before septic metastatic spread [6]. This early recognition and intervention were probably the key contributing factors to our patient's good outcome and survival.

## Conclusion

We report a patient with Lemierre's syndrome resulting from streptococcal induced otitis media and mastoiditis. This is a rare cause of an already rare syndrome that requires prompt recognition and management for patient survival.

## Abbreviations

CT: computed tomography; MRI: magnetic resonance imaging; MRV: magnetic resonance venography.

## Consent

Written informed consent was obtained from the patient's parents for publication of this case report and any accompanying images. A copy of the written consent is available for review by the Editor-in-Chief of this journal.

## Competing interests

The authors declare that they have no competing interests.

## Authors' contributions

CH was the primary author, researched the topic, and organized the paper. MJ and HR admitted and cared for the patient, and followed his hospital course. TF was the principal care physician during the patient's hospital course. SR continues to follow the patient and gives updated information on his status. HN assisted with interpretation of imaging studies. DH assisted with interpretation of imaging and clinical correlations, continues to follow the patient as an outpatient, and provides updates. MK greatly assisted with paper presentation, organization and drafting. AT assisted with interpretation of imaging and clinical correlations. JG, FF and VM researched the topic and gave input on the structure and organization of the paper.
